# Modeling needs user modeling

**DOI:** 10.3389/frai.2023.1097891

**Published:** 2023-04-06

**Authors:** Mustafa Mert Çelikok, Pierre-Alexandre Murena, Samuel Kaski

**Affiliations:** ^1^Department of Computer Science, Aalto University, Espoo, Finland; ^2^Department of Computer Science, University of Manchester, Manchester, United Kingdom

**Keywords:** user modeling, probabilistic modeling, machine learning, human–AI collaboration, AI assistance, human-centric artificial intelligence, human–AI interaction

## Abstract

Modeling has actively tried to take the human out of the loop, originally for objectivity and recently also for automation. We argue that an unnecessary side effect has been that modeling workflows and machine learning pipelines have become restricted to only well-specified problems. Putting the humans back into the models would enable modeling a broader set of problems, through iterative modeling processes in which AI can offer collaborative assistance. However, this requires advances in how we scope our modeling problems, and in the user models. In this perspective article, we characterize the required user models and the challenges ahead for realizing this vision, which would enable new interactive modeling workflows, and human-centric or human-compatible machine learning pipelines.

## Copernician revolution: Quest for objective modeling

**Figure 1:**
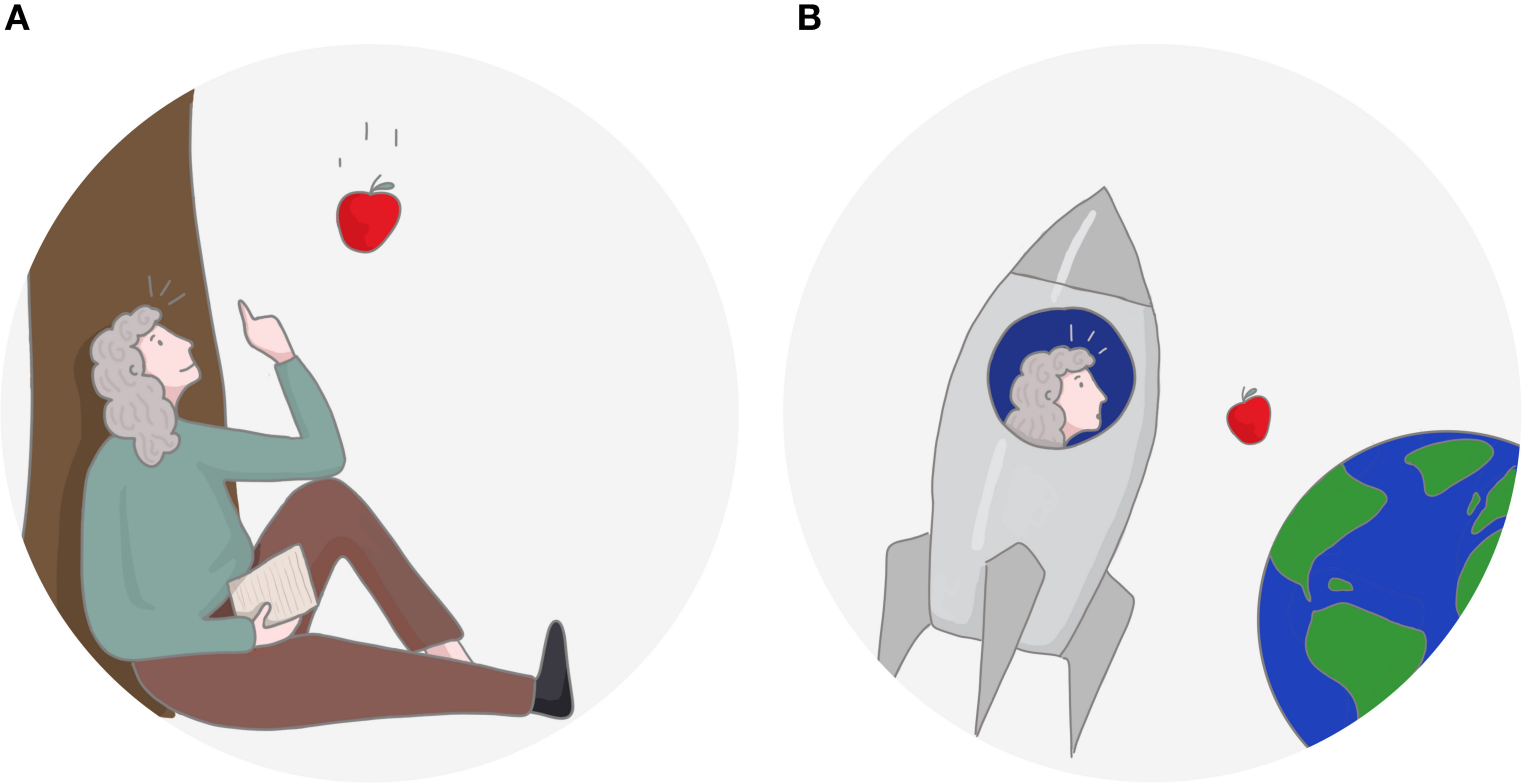
**(A)** Modeling classically aims at an objective description, from which the modeler should be absent. **(B)** However, the choice of the model may depend on the modeler and their experiences; Newton probably would not have proposed the same model of gravity, had he grown up in a space shuttle. We argue that helping a modeler in modeling may require putting the modeler back into the model.

Ever since the Copernican revolution, models have been meant to be objective descriptions of the world, and subjective elements in them are minimized. The mindset in empirical sciences is to form an objective description of a phenomenon, in a mathematical form when possible (Figure 1), from a set of observations. In probabilistic modeling for statistical data analysis, which we mainly focus on, the modeler's beliefs do enter in Bayesian inference, though in scientific data analysis only as domain knowledge (Gelman et al., 2013).

In the current data science mindset, an objective statistical model is inferred from a dataset, and is then ready to be interpreted. However, this simplified mindset, which we brought up to concretize our point, ignores the fact that the data have been collected by someone, often by the modeler, for specific reasons. Without understanding the data collection policy and the reasons, we cannot fully understand the data. For instance, it is not possible to conclude on the efficacy of a treatment by the percentage of cured patients, if only the most severely ill are treated. This leaves us with the following dilemma: the modeling must be objective and not depend on the modeler; yet taking into account the modeler's goal and data collection policy seems necessary as well. We need a new mindset.

## Anti-Copernican revolution with user modeling

### Modeling becomes a human–AI collaboration problem

Recent developments in machine learning make a new mindset possible: the computer can work as a collaborative *assistant*, working together with the human. For modeling, this means being able to assist the human modeler even when they do not yet know the desired outcome well enough to specify it.

When the modeler *is* able to specify their data analysis task precisely, modeling ultimately boils down to a workflow which can be standardized to a reasonable extent (e.g., Bayesian workflow; Gelman et al., 2020). In this case, current computational tools take us a long way. In particular, for model fitting, probabilistic programming languages require the modeler only to specify the model and data, and the tool does the rest.

Conversely, when the task cannot be specified in advance, the modeler must proceed iteratively, by trying out solutions, observing new intermediate results and refining their hypotheses (Gelman et al., 2020). During this process, the modeler gradually understands better the data and the potential computational and statistical issues. The modeler may also need to collect new data. For instance, in personalized medicine, the diagnosis can be sharpened by iteratively developing hypotheses on the etiology, and then refining them after additional examination by the physician. Here, each new observation provides new information and affects the future choices of the modeler.

Unfortunately, there are not many computational tools available that can provide interactive assistance in the long and costly process of iterative modeling. We argue that developing such advanced tools requires including the human–AI collaboration angle in the modeling. Specifically, cooperative assistance can be formulated as a sequential decision-making problem for an AI assistant, whose task is to decide which recommendations to make to the user. For learning, the assistant gets some delayed feedback on the success of the modeling task and instantaneous feedback from the user's reactions to the recommendations. Therefore, it becomes essential for the AI to be able to understand the reasoning behind the user's feedback.

Consider the case of iterative drug design. It is difficult for humans to provide a precise specification of the desired chemical properties of a drug, because even experts do not know all these properties yet, and even if they do, they may only know them implicitly. Current systems force users to specify these chemical properties, and as a result, the specification can be imprecise. Instead, if the users had an advanced AI assistant that is able to interactively suggest good directions to explore the chemical landscape, this would make it easier for them to arrive at precise specifications. It has been shown by Sundin et al. (2022) that pure RL-based solutions give unsatisfactory results, and that even simple human-in-the-loop helps. In the following, we will show how advanced user models can improve the assistance in this case.

In this novel mindset, the task of the AI becomes to assist the modeler by extending the modeler's abilities to reach their goals. This is also known as collaborative intelligence (Epstein, 2015). Then, the modeling process becomes a *human–AI collaboration* problem, in which the AI needs to model the modeler as a part of the process.

### Human–AI collaboration needs advanced user modeling

Collaborative intelligence requires both parties to understand each other. Current modeling engines lack such understandability: it is very difficult to guarantee that the user understands the produced result. This is especially the case with the emerging Automatic Machine Learning (autoML) (He et al., 2021). Black-box approaches such as autoML may be excellent for *automation* (Wang et al., 2019), but we want to *augment* the user's modeling capabilities instead of fully automating modeling. For the outcome where a model is both useful to and understood by the user, it is important to involve the user in the modeling process. It is also crucial that the assistant understands what the user wants and why they prefer certain actions to others, in order to satisfy the goal and preferences of the user.

Just like the AI, the user also develops an understanding of the AI assistant during the collaboration. Humans interpret the actions of other humans as stemming from their goals, and they extend the same interpretation to AI assistants. Thus, the user perceives the actions of the AI as stemming from a goal and interprets the assistant's recommendations accordingly. Then to understand the user's response, the AI needs to understand how the user interprets its actions. This recursive understanding of each other is characteristic of any collaboration. It makes the cooperation possible by ensuring that the two agents can fully benefit from the other's actions.

Even though it may sound counter-intuitive for our initial goal of developing objective models, what we exposed now shows that the next step in advanced modeling actually requires including a model of the modeler into the modeling process. This is not inconsistent with objectivity of modeling, once we realize that the traditional modeling mindset is restricted to cases where the goal of modeling is fully specified. Including the modeler in the model enables objective modeling also when the goal is tacit, by inferring it interactively. If the modeling is done properly, the modeling process remains equally objective and for the goals of the modeler, the end result is the same as if they had been able to specify the goal fully in advance.

## Next-generation user models

User models have a long history in machine learning systems such as recommender systems and information retrieval engines. However, existing works regard the user mostly as a *passive* agent *reacting* to their environment, for instance accepting or rejecting recommendations based on their tastes. What has been missing is a vision considering the user as an active decision-maker engaged in a two-agent interaction with an AI collaborator. How to endow an artificial agent with advanced models of the user remains an open question, which is at the core of human–AI cooperation research. The challenge is to find a simple enough model of the user to be learnable, while still being useful to the user. Such a model needs to take into account at least **three key characteristics**, detailed in the next subsections.

### The user has a goal

The AI assistant first needs to understand that the user *has* a goal, and eventually what this specific goal is. The user typically does not select their actions by responding passively, but actively plans to reach their goal. The modeling decisions made by a modeler are all motivated by a purpose, which in research can be as general as to understand a phenomenon in a faithful way. In drug design, it is to find a molecule with specific properties, which the designer is not able to fully explicate.

Indeed, in many cases, the user may not be able to specify their goal, either because they do not yet know what is feasible, or they are not able to articulate the desired outcomes. The user may also simply prefer to save the effort and get help in specifying all details. Every piece of detail left unspecified, or imprecisely specified, opens the door to shortcut solutions which the user may not want.

Hence, the AI assistant needs to infer the user's goal through the interaction. By observing the actions of the user, the assistant can attempt to infer what objective the user is maximizing and what goal is being pursued. This is the task of Inverse Reinforcement Learning (IRL) (Ng and Russell, 2000). Alas, IRL is inherently limited, in particular by unidentifiability issues and requirements it places on the quality of the user input.

### The user has a limited cognitive computational capacity

Knowing that the user has a goal is not enough; it is also essential to know how they plan on reaching this goal. This is important to be able to anticipate properly the actions of the user, and to infer the goal correctly.

Indeed, it is impossible to infer the reward being maximized, if the maximization process is not known (Armstrong and Mindermann, 2018). This corresponds to understanding the limitations and constraints of the agent. An agent with infinite resources could base its choices on the exploration of *all* possible sequences of outcomes. Such an exploration is obviously computationally prohibitive when planning over long horizons.

In this sense, a human's behavior depends on their cognitive computational capacity: we search the optimum among only a subset of scenarios, selected according to our cognitive computational capabilities. For instance, we may ignore scenarios that we deem too improbable. This idea of being rational within the bounds of a given computational capacity and other constraints, is known as *computational rationality* (Lewis et al., 2014; Gershman et al., 2015). As an example, when incrementally designing a molecule, a user having expertise on specific types of molecules and currently considering incremental changes in them, may not want to be distracted by completely different kinds, even if they are theoretically optimal in some sense.

It becomes then difficult for an AI assistant to anticipate the user's actions and to infer their goal, if these bounds are not identified. Therefore, the AI must have a model of the user as a computationally rational goal-seeking agent. However, the nature of a human's cognitive bounds is very different from the bounds usually implemented in an artificial agent, and prior knowledge needs to be brought from cognitive sciences, psychology, and human-computer interaction which study the specifics of human reasoning.

### The user has a theory of the assistant's mind

No matter how good the plan of the assistant is, the user will perceive and interpret it from their own point of view (Chakraborti et al., 2020) and will accept a suggestion of the assistant only if they consider it as useful to reach their goal.

Humans tend to interpret actions of others as goal-oriented, as part of a plan to reach their goals, and that is what the user is likely to do for an AI-assistant's actions as well: the user models the system and builds a *theory of the AI's mind* (Chandrasekaran et al., 2017). If the user can decipher the AI's strategy, they can assess whether it aligns with their own, and if it does, are more likely to accept the AI's suggestions and converge to their goal more quickly. This implies the AI needs to model the user at a more advanced level: as having a model of the assistant. Recent works have demonstrated the efficiency of models of this type (Peltola et al., 2019).

**Figure 2:**
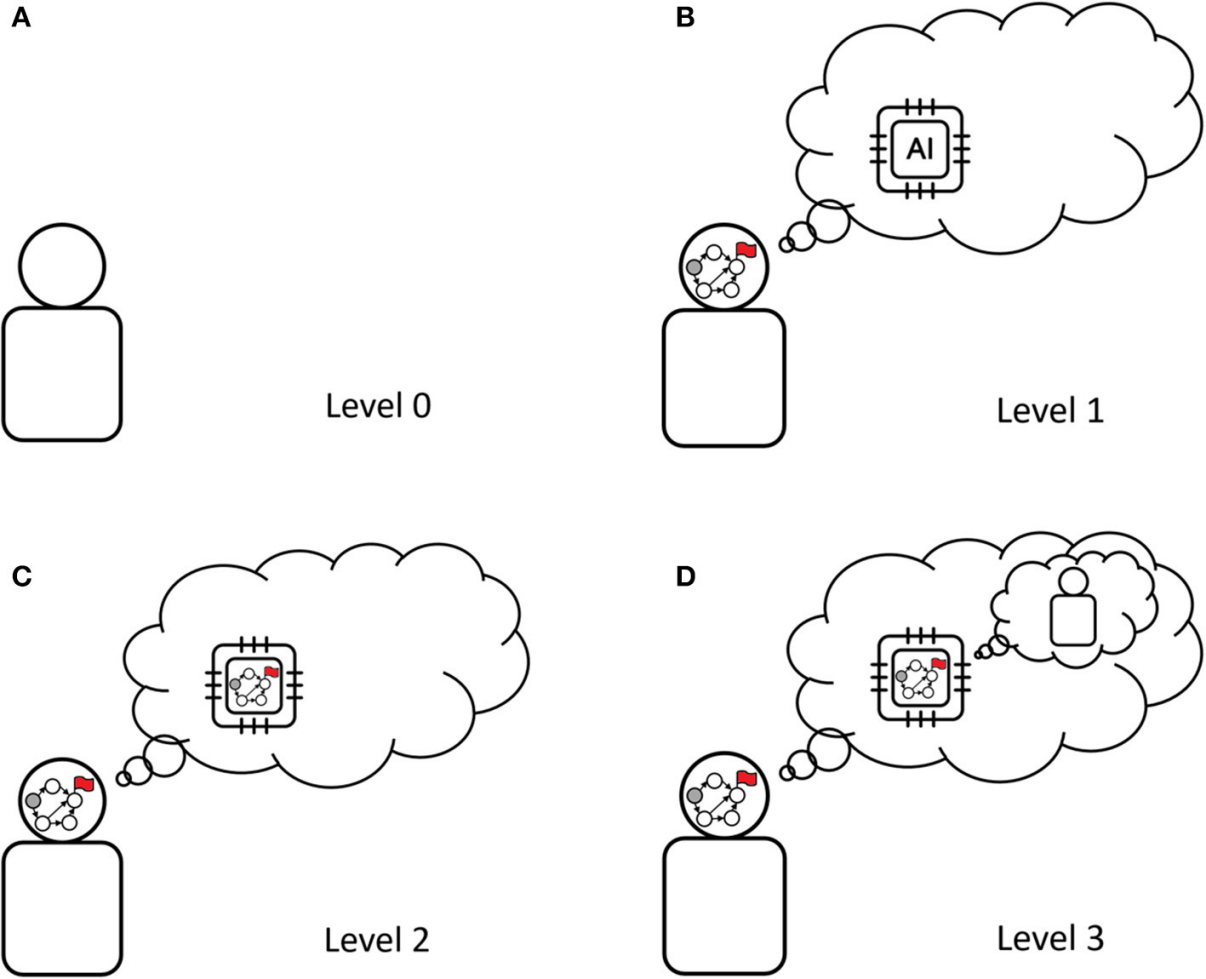
Four levels of user models. Each level corresponds to the AI assuming a different mode of decision-making of the user. An AI system having a higher-level user model is able to take into account different types of user's reasoning. The graph in the head of some users and in the AI's chip denotes planning capability: it is a planning graph with the red flag signifying a goal. The AI having access to a higher-level user model means that the AI has more advanced user modeling capabilities, accounting for the user reasoning about the AI. From left top to right bottom: **(A) Level 0**: the user is not pursuing a goal, **(B) Level 1**: the user is pursuing a goal, but does not consider the AI is pursuing one as well, **(C) Level 2**: the user is pursuing a goal and understands that the AI is pursuing a goal too, **(D) Level 3**: the user is pursuing a goal, understands that the assistant is doing the same, and that the assistant will interpret user actions as goal-pursuant.

In summary, we considered multiple *levels* of understanding encoded into the user model (Figure 2), inspired by the *level-k* (Stahl and Wilson, 1995; Bacharach and Stahl, 2000) and *cognitive hierarchy* (Camerer et al., 2004) theories from behavioral game theory. They describe how the AI assistant understands the user. Up to now, we have described three levels:

**Level 0: The user does not pursue a goal**. This is the minimal model we may consider, and is used by most systems nowadays, in particular by recommender systems. An assistant endowed with such a model does not aim to understand the goal pursued by the user, and treats them as a passive data source. For instance, a drug design assistant would suggest modifications that are optimal in its opinion.

**Level 1: The user is pursuing a goal, but does not consider the AI to be goal-pursuant**. An assistant endowed with a level 1 model will make suggestions that are optimal to the user, but which may be not understood as such by the user. A drug design assistant would try to understand the user's goal and make suggestions that are optimal in the sense of this goal.

**Level 2: The user is pursuing a goal and understands that the AI is pursuing a goal too**. These models can guarantee better understandability of the assistant's actions, by enabling the assistant to take into account how the user would perceive its objectives. A drug design assistant could visualize a molecule such that the user understands why the design change is actually useful for the user's goal.

Following this logic, a **level 3 user model** would then consider a user who is pursuing a goal, understands that the assistant is doing the same, and understands that the assistant will interpret user actions as goal-pursuant. In their seminal work, Wright and Leyton-Brown (2020) define two critical aspects for an agent to be considered strategic: it must adapt its behavior to the goals of others (*other-responsiveness*), and it must also pursue its own goals (*dominance responsiveness*). In Figure 2, only user models that are level-2 or above can be considered strategic. For instance, in video recommendation, a strategic user will not choose to click “like” based on their taste only, but also by anticipating the influence such a click will have on the future video recommendations by the AI. When users exhibit complex strategic behaviors, models below a certain level cannot explain or fit their behavior.

## Challenges in learning user models

The main motivation for having a user model is to personalize the assistance in order to help the user more effectively. The parameters of the user models need to be inferred separately for each user and their task, based on observed behavior while interacting with them. More specifically, user models act as likelihood functions, and are combined with informative priors and models of the data-analysis task, when inferring of the models of specific users carrying out a specific task.

Here, we present open challenges of user model inference for AI-assistance, in order to sketch the necessary research directions.

### Small data

Learning the parameters of the user model online, from the user's interaction with the assistant, is challenging due to data scarcity. Collecting more data may be too costly, or even impossible, as it requires exploratory actions which may not be helpful for the user.

The data scarcity makes informative priors necessary. The priors can come in the form of carefully designed user models that generalize the existing results of behavioral sciences into AI-assistance (Griffiths and Ho, 2022). Interaction data of other users can be integrated using hierarchical models (Kruschke and Vanpaemel, 2015). However, users' data are particularly sensitive and cannot be exchanged carelessly, and simply removing identifiers is not sufficient to preserve privacy (Narayanan and Shmatikov, 2008). Therefore, advanced privacy-preserving techniques must be developed for data sharing between users.

### User behavior is non-stationary

The user learns and changes their behavior; their vision is refined along the process, as they acquire a more accurate understanding of the task and environment, and their goals might change over time.

Such changes can be seen as *distributional shift* (Quiñonero-Candela et al., 2008), where the data distribution changes over time, or as an example of agent non-stationarity (Hernandez-Leal et al., 2017) in a multiagent context. One important challenge here is to identify and predict these changes. This corresponds to modeling the evolution of the parameters of the user model, which is difficult due to the extensive uncertainty and partial observability of this evolution.

### User models require heavy computation

Realistic user models may be computationally complex. This is an issue for standard approaches to probabilistic inference, such as Monte Carlo methods, which require a likelihood function that is computationally easy to evaluate. This issue appears even with elementary user models: for instance, if we model a user as a decision-maker who plans *T* steps into the future, the assistant will have to solve a *T*-step planning problem for each evaluation of the likelihood function. Things get even worse when considering higher-level user models such as level-2 and level-3, requiring recursive reasoning.

A promising direction is likelihood-free inference, which circumvents the need to define the likelihood function explicitly. In particular, approximate Bayesian computation (ABC) can be done based on comparing the observed data to synthetic data generated using parametric surrogate models (Sunnåker et al., 2013). This comparison critically relies on the choice of relevant summary statistics, and this choice for user models is still an open problem. Additionally, for user modeling, ABC needs to be extended to cope with the non-stationarity discussed in Section 4.2.

### User models may not be learnable

Complex user models can capture more subtle user behaviors, but they may not be learnable due to unidentifiability. For instance, the goal and the bounds of an agent cannot in general be inferred simultaneously (Armstrong and Mindermann, 2018). Unidentifiability worsens when considering higher-level models, in which the user considers the consequences of their own actions on the assistant's behavior. For instance, a user might reach for something because this is their goal, or because they think this will lead the AI to help them with their actual goal.

An incorrect user model may lead to wrong predictions of the user's behavior, which would make AI assistance inadequate at best. Informative priors and carefully designed model families can help with unidentifiability; however, choosing simpler model families can increase the risk of model misspecification. Therefore, it is crucial to analyze the relationship between assistance and the learnability qualities and expressivity of user models (Bajcsy et al., 2021).

## Conclusion

Modern machine learning systems can and have been argued to be necessary tools for solving the current and future grand challenges. These problems increasingly lie at the frontier of human knowledge, where we cannot specify our goals and values explicitly. Therefore, the stakes are getting higher for developing human-centric AI solutions that are able to collaboratively help us. This paper is a call to arms, in which we argue that post-hoc approaches to making machine learning systems human-centric are not enough. The next generation machine learning systems need AI-assistants that can interactively help both system designers and end users reach even tacit and evolving goals. Such AI-assistants must model their human users to reach mutual understanding and meaningful interaction. We have argued that this requires making the human an integral part of the model and hence the machine learning system itself, rendering it directly human-centric. We identified main directions for developing such AI-assistants, where much more research is required.

## Data availability statement

The original contributions presented in the study are included in the article/supplementary material, further inquiries can be directed to the corresponding author.

## Author contributions

All authors listed have made a substantial, direct, and intellectual contribution to the work and approved it for publication.
